# Evaluation of Spontaneous Pneumothorax Surgeries: A 16-Year Experience in Japan

**DOI:** 10.1155/2016/7025793

**Published:** 2016-04-13

**Authors:** Ryo Takahashi

**Affiliations:** ^1^Department of General Thoracic Surgery, National Hospital Organization Chiba-East Hospital, Chiba 260-0856, Japan; ^2^Department of General Thoracic Surgery, Graduate School of Medicine, Chiba University, Chiba 260-0801, Japan; ^3^Department of Respiratory Medicine, Jinken Clinic, Kanagawa 243-0432, Japan

## Abstract

*Background.* Video-assisted thoracoscopic surgery is the surgical procedure of choice for spontaneous pneumothorax due to its noninvasiveness and convenience. A higher recurrence rate with thoracoscopic bullectomy (TB) than that after traditional thoracotomy (TT) led us to adopt thoracoscopic double-loop ligation (TLL) as our standard procedure in 1998. This study compares the effectiveness and safety of these 3 operative procedures.* Methods.* Patients who underwent their first surgery for spontaneous pneumothorax at our hospital between January 1994 and December 2010 were included. Patients with a history of surgery for spontaneous pneumothorax, those with special clinical conditions such as lymphangioleiomyomatosis, or those with catamenial, traumatic, or iatrogenic pneumothorax were excluded.* Results.* A total of 777 males (14–91 years old; 814 pneumothorax sides), and 96 females (16–78 years old; 99 pneumothorax sides) were included in the study. TT was performed in 137 patients (143 sides), TB in 106 patients (112 sides), and TLL in 630 patients (658 sides). The postoperative recurrence rates were 3.5%, 16.1%, and 5.3% in the TT, TB, and TLL groups, respectively (*p* < 0.0001). Mean blood loss and operating time were lowest for TLL.* Conclusions.* The results suggest that TLL should be the surgical procedure of choice for spontaneous pneumothorax.

## 1. Introduction

Spontaneous pneumothorax, a benign, self-limiting condition, is currently treated using conservative options, like chest-tube drainage, or surgically, by thoracotomy and video-assisted thoracoscopic surgery (VATS) [1–3].

Compared to thoracotomy, VATS has benefits of less postoperative pain, better wound cosmetics, shorter hospital stay and duration of drainage, better functional recovery, better short- and long-term patient satisfaction, and equivalent cost-effectiveness [2, 4–6]. Additionally, it is associated with negligible mortality and fewer postoperative complications [2]. Although the postoperative recurrence rate of pneumothorax following VATS remains higher than that after traditional thoracotomy [7, 8]. VATS is recommended in patients with a first episode or recurrent spontaneous pneumothorax due to its noninvasiveness and convenience [2, 4, 9–12]. Various surgical techniques such as conventional suturing, electrocautery ablation, endoscopic stapling and resection (thoracoscopic bullectomy), neodymium-yttrium-aluminum-garnet laser ablation, and endoloop ligation of bleb or bulla can be carried out using VATS for the treatment of spontaneous pneumothorax [2, 13].

Among the different VATS-assisted procedures currently available, endoloop ligation is particularly useful in patients with diffuse emphysematous lung disease or giant bullae wherein the base of the bullae is too broad for placement of the stapler during endoscopic stapling [9]. Specifically, this technique allows the collapsed thin emphysematous lung parenchyma in such patients to be successfully ligated [9, 13–17]. The endoloop ligation technique is considered the treatment of choice when spontaneous pneumothorax manifests itself intraoperatively as multiple smaller blebs [14, 15].

The National Hospital Organization Chiba-East Hospital, Japan, initiated thoracoscopic bullectomy as an alternative to traditional thoracotomy in 1994. The results obtained were consistent with those of other studies [7, 8]; specifically, a higher recurrence rate was observed than that after thoracotomy. Therefore, thoracoscopic double-loop ligation of the bullae was started as a standard, alternative procedure in 1998. The present study compared the effectiveness and safety of 3 operative procedures for spontaneous pneumothorax—thoracoscopic bullectomy, thoracoscopic double-loop ligation, and thoracotomy. Specifically, the postoperative recurrence rates of these surgical procedures were compared to evaluate the potential of thoracoscopic loop ligation as the treatment of choice for spontaneous pneumothorax.

## 2. Materials and Methods

Approval for this study was given by the ethics committee of the National Hospital Organization Chiba-East Hospital, Chiba, Japan.

### 2.1. Inclusion and Exclusion Criteria

**Figure 1:**
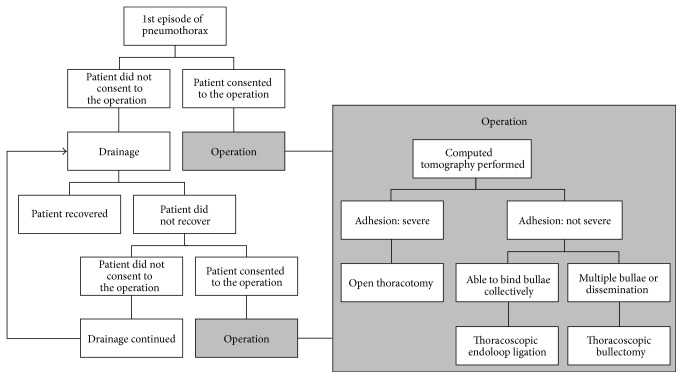
Decision schema for choosing the surgical procedure (CT: computed tomography; VATS: video-assisted thoracoscopic surgery).

This retrospective study included patients who underwent their first surgery for spontaneous pneumothorax at the National Hospital Organization Chiba-East Hospital, Chiba, Japan, between January 1994 and December 2010. Patients were surgically treated when conservative management failed or was not indicated. Patients with a history of surgery for spontaneous pneumothorax, those in whom surgery or general anesthesia was contraindicated, or those with other types of pneumothorax such as catamenial, traumatic, or iatrogenic pneumothorax were excluded from the study. For patients initially treated with pleural drainage, surgical treatment was recommended (Figure 1) as per standard guidelines if the leak persisted after 1 week [18]. This study was conducted in accordance with Good Clinical Practice guidelines and the ethical principles evinced in the Declaration of Helsinki.

### 2.2. Choice of Surgical Procedure

A preoperative conventional computed tomography (CT) scan at 2 to 5 mm intervals was performed to assess the underlying disease. The choice of surgical procedure was based on the site, shape, number of the cysts, degree of lung collapse, and a desmoplastic range determined preoperatively and reevaluated during surgery. In general, after 1998, thoracoscopic double-loop ligation was the surgical technique of choice unless contraindicated. If the adhesions were not severe and it was possible to bind the bullae collectively (e.g., several cysts in the apex of the lung), thoracoscopic double-loop ligation was carried out. However, when ligation was not possible, as in cases with a wide-based cyst, cysts ranging in a comb form, or with multiple bullae or dissemination, a thoracoscopic bullectomy was carried out. The VATS was converted to open thoracotomy only if continuation of the VATS was judged to be difficult during the surgery, as in the case of severe adhesions, dissemination, or emphysematous lesions.

**Figure 2:**
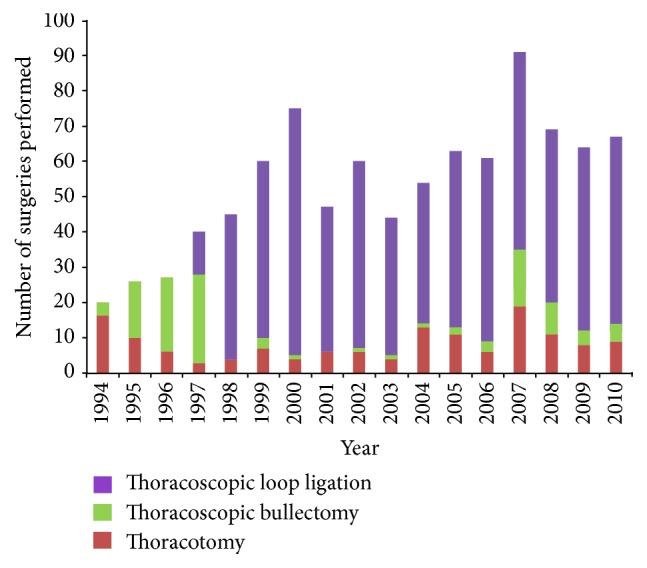
Distribution of thoracotomy, thoracoscopic bullectomy, and thoracoscopic loop ligation performed at the National Hospital Organization Chiba-East Hospital between January 1994 and December 2010.

Patients that presented with their first episode of spontaneous pneumothorax between 1994 and 1997 were primarily operated on via thoracoscopic bullectomy. However, from 1998 to 2010, thoracoscopic loop ligation was the treatment of choice. The distribution of thoracotomy, thoracoscopic bullectomy, and thoracoscopic loop ligation during the study period is presented in Figure 2.

### 2.3. Surgical Procedures

**Figure 3:**
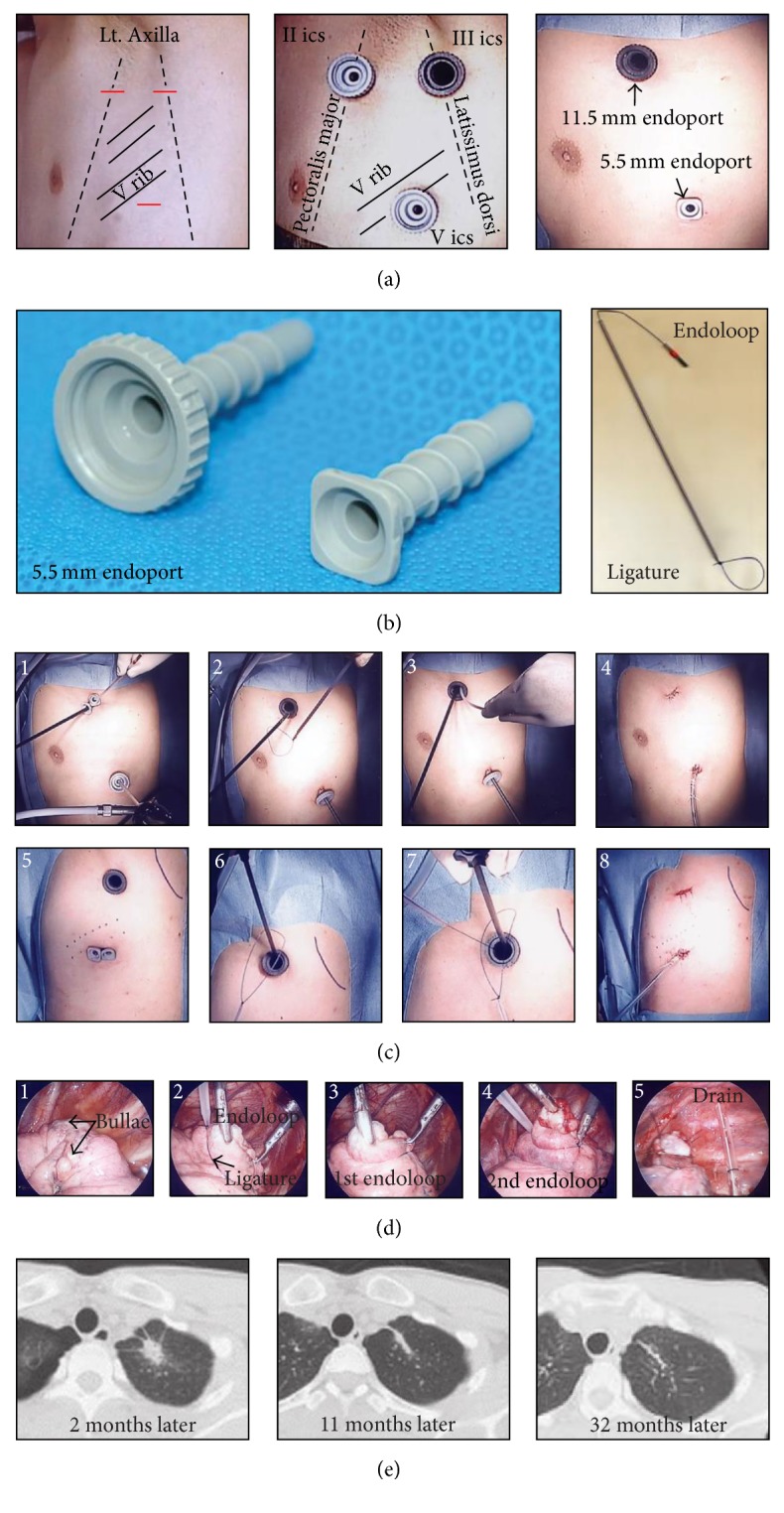
Schematic of the thoracoscopic endoloop ligation procedure. (a) Thoracoscopic ports placed on the anterior axillary line in the second intercostal space and midaxillary line in the fifth intercostal space. (b) Surgical instruments. (c) External view of the operative procedure images. (d) Thoracoscopic view of the procedure. (e) CT scan images at 2, 11, and 32 months after a successful operation.

VATS was performed under general anesthesia in the lateral position using differential lung ventilation. A thoracoscopic port was placed on the anterior axillary line in the second intercostal space and midaxillary line in the fifth intercostal space, in principle. For the thoracoscopic loop ligation procedure, Endoloop PDS-II0 ligature (Ethicon, USA) was used for the double-loop ligation of the bullae (Figure 3). The endoloop was used to ligate the outer surface of the bullae in order to shrink them and reduce the cavity size to a minimal extent. This helped in expanding the adjacent lung tissue and improving lung function. For the thoracoscopic bullectomy procedure, an Endo GIA*™* Autosuture device (Covidien, USA) was used.

### 2.4. Follow-Up

After discharge, follow-up was scheduled at 1 week, 1 month, 6 months, and 1 year at the outpatient department of the hospital. Almost all cases were followed up for 1 year after surgery. Follow-up was continued until 5 years in some patients to assess postoperative anastomosis. Patients were advised to report to the hospital in case they had breathing difficulties at any time during the follow-up and also after the final follow-up.

### 2.5. Statistical Analysis

The data were tabulated and analyzed. Chi-square test was used to compare recurrence rates after surgical procedures; two-sided *p* < 0.05 was considered statistically significant. The analyses were conducted using JMP 10.0.2 from SAS (SAS Institute Inc., Cary, North Carolina, USA).

## 3. Results

Of the 873 patients included in the study, 777 were males (age range: 14–91 years) and 96 were females (age range: 16–78 years). A total of 913 sides (777 male, 96 female, and 40 bilateral) were operated upon, of which thoracotomy, thoracoscopic bullectomy, and thoracoscopic loop ligation were carried out in 15.7%, 12.3%, and 72.1% of cases, respectively. The baseline characteristics of patients who underwent any of the 3 operative procedures are presented in Table 1.

At baseline, the thoracoscopic bullectomy group had a higher mean number of bullae (1.52) compared to thoracotomy (1.15) and thoracoscopic endoloop ligation (1.12) groups. The proportion of patients with more than 2 (range: 3–6) bullae at baseline was also the highest (20.5%) in the thoracoscopic bullectomy group, compared with the thoracotomy (7.7%) or the thoracoscopic endoloop ligation (3.0%) groups. Emphysema was present in nearly one-third (28%) of the cases referred for thoracotomy, whereas it was present in approximately 10% of the cases in the other 2 surgical groups. Nearly 45% of patients in the thoracotomy group were 60 or older.

The mean operating time was the lowest in the thoracoscopic ligation group compared to the other groups. Additionally, blood loss was significantly less in patients treated with thoracoscopic endoloop ligation versus the other groups (Table 2).

No intraoperative complications were reported in any of the groups. Patients were followed up for between 1 and 3 years. Overall, the postoperative pneumothorax recurrence rate, a major postoperative complication, was the highest in the thoracoscopic bullectomy group (16.1%) followed by the thoracoscopic endoloop ligation (5.3%) and thoracotomy (3.5%) groups (*p* < 0.0001) (Table 3). A similar trend was observed in the analysis of the age-stratified recurrence rates.

The results showed that almost 40% of the surgeries were performed in patients below 20 years of age. The recurrence rate was the highest in the same age group and decreased with increasing age. The thoracoscopic bullectomy group had higher recurrence for almost all age categories, followed by thoracoscopic loop ligation and thoracotomy.

The mean duration of recurrence to pneumothorax after surgery was 10.6, 15.5, and 16.2 months in the thoracotomy, thoracoscopic bullectomy, and thoracoscopic loop ligation groups, respectively. Thus, a longer recurrence-free time interval was observed in the thoracoscopic loop ligation group.

Apart from recurrence, the highest overall postoperative complication rates were observed in the thoracotomy group (12.6%) followed by the endoloop ligation group (4.3%) and the thoracoscopic bullectomy group (3.6%) (*p* = 0.0002). The most common postoperative complication was continuing leakage observed in 9 (6.3%) patients in the thoracotomy group, 3 (2.7%) in the thoracoscopic bullectomy group, and 13 (1.9%) in the thoracoscopic endoloop ligation group (*p* = 0.0164). In the thoracotomy group, drug allergy, pneumonia, gastric ulcer, and pneumonia/continuing leakage were observed in 1 patient each (0.7%), whereas heart failure was observed in 2 patients (1.4%). In the thoracoscopic endoloop ligation group, pneumonia was observed in 3 patients (0.46%), whereas pulmonary infarction, acute renal failure, and cerebral infarction were observed in 1 patient each (0.15%). Sliding of loops in 7 patients (0.1%) in the endoloop ligation group required reoperation. The sliding occurred 1–3 days after surgery, and patients presented with sudden respiratory discomfort; all patients with sliding of loops underwent thoracotomy.

In the thoracotomy group, 2 patients died due to pleural hemorrhage and respiratory failure 4 weeks after the operation.

## 4. Discussion

Our results show that thoracoscopic endoloop ligation was an effective and safe procedure with minimal complications in patients who received their first surgery for spontaneous pneumothorax. The recurrence rate after thoracoscopic endoloop ligation (5.3%) was significantly lower compared to that after thoracoscopic bullectomy (16.1%), and similar trends were observed in the age-stratified analyses. Further, it had shorter operating time and less blood loss compared with both thoracoscopic bullectomy and thoracotomy. Thus, thoracoscopic endoloop ligation demonstrates a potential to become the surgical procedure of choice for patients with spontaneous pneumothorax.

Thoracoscopic endoloop ligation of bullae has been previously used successfully in patients with bullous emphysema and spontaneous pneumothorax [9, 13, 14, 19–21]. However, to the best of our knowledge, this is one of the largest case series from Japan (873 Japanese patients presenting with their first episode of spontaneous pneumothorax) that shows that thoracoscopic endoloop ligation is safe and effective.

Thoracoscopic endoloop ligation is regarded as an operative method of first choice in our hospital. We also use endoloop ligation for the reinforcement of the resection line stump of the automatic suture instruments. In our experience, the endoloop ligature is suitable for the majority of cysts, including cysts with fistulae, and has an added advantage of avoiding neogenesis of the bullae. Young individuals with primary spontaneous pneumothorax often show a predilection to cyst colonization with narrow base, wherein endoloop ligation may be used. Thus, the proportion of thoracoscopic endoloop ligation was significantly higher in the present case series.

In many hospitals, linear stapling devices are preferred for resection of bullae during VATS for treatment of spontaneous pneumothorax, despite being more expensive [21, 22]. This can be attributed to the safety, convenience, and possibility of identifying underlying disease that is associated with their use [14, 17, 21]. Thoracoscopic endoloop ligation of bullae can be a good alternative to bullectomy using endostapling devices, as it has been shown to be cost-effective, in addition to being minimally invasive, safe, simple, and ubiquitously available [13, 14, 21, 22]. Given the lower recurrence rates observed with endoloop ligation versus thoracoscopic bullectomy in our study, it is reasonable to support the use of endoloop ligation for parenchymal bullae ligation in patients with spontaneous pneumothorax, especially in healthcare centers with limited medical budgets [13, 21].

A few large studies, with a postoperative follow-up period similar to ours, showed a slightly lower recurrence rate (range: 1.3–2.1%) [20, 22, 23], especially for thoracoscopic bullectomy, compared with the current results. The reasons for the higher recurrence rate of VATS observed in our study may include differences in patient population, surgical techniques, and/or surgeon experience.

Liu and colleagues evaluated the long-term effect of endoloop ligation compared with staple bullectomy [21]. A series of 226 patients who had been surgically treated for primary spontaneous pneumothorax (130 with endoloop ligation and 96 with staple bullectomy) were retrospectively analyzed. Interestingly, similar to our results, a significantly lower recurrence rate was observed in the endoloop ligation group compared with the staple bullectomy group (6.2% versus 17.7%; *p* = 0.006) [21]. However, the limited evidence in the literature comparing the relative benefits of endoloop ligation* vis-à-vis* thoracoscopic bullectomy in comparable patient cohorts for the prevention of pneumothorax recurrence precludes generalization of these results. Further randomized controlled trials that compare these surgical procedures are needed.

The mean operating time observed during VATS in our study (thoracoscopic bullectomy: 86 minutes; thoracoscopic endoloop ligation: 61.32 minutes) was comparable to that reported in previous studies (average range: 40–100 minutes) [13–15, 20, 21]. The lower operating time and blood loss observed during endoloop ligation compared to other techniques in the current study provide evidence that this may be considered the treatment of choice in patients in whom invasive surgical options are contraindicated.

Epidemiologic studies reveal that the peak incidence of primary pneumothorax occurs in young individuals, whereas that of secondary pneumothorax occurs in individuals above 55 years old [24]. With approximately 70% of the patients in our treatment cohort being ≤40 years of age, our study probably represents more cases of primary than secondary spontaneous pneumothorax.

It is well known that primary spontaneous pneumothorax occurs more frequently in males; however, the male : female ratio varies considerably in different studies reporting surgical management of pneumothorax—from approximately 3 : 1 to 6 : 1 [24]. Our case series included 88% males and 12% females. The higher proportion of men in our study may have resulted from the exclusion of patients with catamenial pneumothorax at baseline.

Despite growing evidence to show that VATS is the procedure of choice for patients with recurrent spontaneous pneumothorax, the likelihood of extending immediate VATS intervention to patients presenting with their first episode of spontaneous pneumothorax still depends on the surgeon's familiarity with the procedure [25]. As this is a benign disease and the recurrence rates are comparable after open thoracotomy (3.5%) and endoloop ligation (5.3%), both are valid surgical procedures. Foroulis and colleagues evaluated the long-term outcome for spontaneous pneumothorax patients after thoracoscopic intervention and axillary minithoracotomy and report that recurrence rates after both procedures are similar [26].

The overall complication rate observed in the endoloop ligation group (4.3%) in the current study was lower than that in earlier studies in patients with spontaneous pneumothorax (range: 6.9–15.2%) [14, 21, 22]. A known complication of endoloop ligation is the accidental slipping off of the loop during lung expansion or after a forceful sneeze. The problem can be minimized by the placement of a double or triple loop around each bulla [9, 27], as was done in the present study. We believe that, as a result, only 7 incidences of the loop slipping off were reported in our study.

The nonavailability of data on smoking, adhesions, and size of bullae for analyses is a drawback of the current study, as these factors could have had an impact on treatment outcome. Additionally, the decision on the type of surgery to be performed was based on the physician's discretion instead of a random assignment. This may be regarded as a study limitation, as it prevents direct comparisons between the recurrence rates for the different interventions. Nevertheless, our study, being the largest case series assessing the comparative effectiveness and safety of these 3 operative procedures in Japanese patients, provides highly relevant data in this disease population.

## 5. Conclusions

This large case series of more than 873 (913 sides) Japanese patients shows that thoracoscopic endoloop ligation is an effective and safe procedure with minimal complications for treating spontaneous pneumothorax. With shorter operating time, less blood loss, and recurrence rates lower than thoracoscopic bullectomy, thoracoscopic endoloop ligation can be used as the treatment of first choice in similar patient populations.

## Figures and Tables

**Table 1:** Baseline characteristics.

Characteristic	Open thoracotomy (*n* = 143)	Thoracoscopic bullectomy (*n* = 112)	Thoracoscopic loop ligation (*n* = 658)
Age (range, years)			
Males	15–87	14–78	14–91
Females	17–78	17–47	14–78
Sex, *n* (%)			
Males	131 (91.61)	100 (89.29)	582 (88.45)
Females	12 (8.39)	12 (10.71)	76 (11.55)
Number of bullae (mean [min., max.])	1.154 [1, 6]	1.518 [1, 6]	1.123 [1, 4]
Emphysema, *n* (%)	40 (27.97)	12 (10.71)	47 (7.14)

*n*: number of sides operated upon.

**Table 2:** Mean operating time and the blood loss during each of the 3 operating procedures.

	Open thoracotomy (*n* = 143)	Thoracoscopic bullectomy (*n* = 112)	Thoracoscopic loop ligation (*n* = 658)
Operation time (minutes)			
Mean (min., max.)	118.37 (42, 201)	86.00 (40, 216)	61.32 (18, 198)
Bleeding (mL)			
Mean (min., max.)	62.34 (1, 620)	9.14 (1, 101)	5.04 (1, 103)

*n*: number of sides.

**Table 3:** Distribution of recurrence rates by age.

	Age (years)					Total
	<20	20 to <40	40 to <60	60 to <80	≥80	
Thoracotomy (*n* = 143)						3.5% (5/143)

Thoracoscopic bullectomy (*n* = 112)	32.3% (10/31)	11.5% (6/52)	0% (0/21)	25% (2/8)	0% (0/0)	16.1% (18/112)

Thoracoscopic loop ligation (*n* = 658)	8.3% (17/206)	4.4% (14/318)	5.3% (4/76)	0% (0/49)	0% (0/9)	5.3% (35/658)

*n*: number of sides.
